# Face recognition: a model specific ability

**DOI:** 10.3389/fnhum.2014.00769

**Published:** 2014-10-10

**Authors:** Jeremy B. Wilmer, Laura T. Germine, Ken Nakayama

**Affiliations:** ^1^Department of Psychology, Wellesley CollegeWellesley, MA, USA; ^2^Psychiatric & Neurodevelopmental Genetics Unit, Massachusetts General HospitalBoston, MA, USA; ^3^Department of Psychology, Harvard UniversityCambridge, MA, USA

**Keywords:** specific ability, individual differences, face recognition, intelligence, IQ, multiple intelligences, cambridge face memory test, generalist gene

## Abstract

In our everyday lives, we view it as a matter of course that different people are good at different things. It can be surprising, in this context, to learn that most of what is known about cognitive ability variation across individuals concerns the broadest of all cognitive abilities; an ability referred to as general intelligence, general mental ability, or just *g*. In contrast, our knowledge of *specific abilities*, those that correlate little with *g*, is severely constrained. Here, we draw upon our experience investigating an exceptionally specific ability, face recognition, to make the case that many specific abilities could easily have been missed. In making this case, we derive key insights from earlier false starts in the measurement of face recognition’s variation across individuals, and we highlight the convergence of factors that enabled the recent discovery that this variation is specific. We propose that the case of face recognition ability illustrates a set of tools and perspectives that could accelerate fruitful work on specific cognitive abilities. By revealing relatively independent dimensions of human ability, such work would enhance our capacity to understand the uniqueness of individual minds.

## Introduction

Most of what we know about human cognitive ability—and by ability, we mean *variation across individuals* in performance or potential—concerns *g*. *g* is the single, broad ability that has been observed to account for a large portion of the variation in any sufficiently large and diverse battery of cognitive tests (Spearman, 1904; Carroll, 1993; Jensen, 1998). Studies of *g* (and of highly g-related tests) have long dominated the human abilities literature, producing the bulk of known genetic (Plomin et al., 2013), neural (Deary et al., 2010), clinical (American Psychiatric Association, 2013), academic (Neisser et al., 1996), professional (Schmidt and Hunter, 2004), and personal (Jensen, 1998; Deary, 2012) correlates of human abilities. In contrast to the literature on *g*, the parallel literature on specific abilities, those abilities that correlate little with *g*, is tiny (Neisser et al., 1996; Jensen, 1998; Schmidt and Hunter, 2004; Deary, 2012).

Why do we know so little about specific abilities? Lack of interest cannot account for this limited knowledge. Theories hypothesizing consequential specific ability dimensions have enjoyed wild popularity in fields as diverse as education and business, as well as in the media (Goleman, 1995; Gardner, 2006). Another possible explanation for the lack of knowledge about specific abilities is that they simply do not play a very important role in our lives (Schmidt and Hunter, 2004). Indeed, upon cursory examination, the sheer size and apparent comprehensiveness of the human abilities literature make it difficult to imagine that important specific abilities could have been missed.

We will argue here, nevertheless, that it is too early to write off specific abilities as unimportant or inconsequential. We propose, on the contrary, that the lack of emphasis on specific abilities is an artifact of (a) traditional test development procedures in the human abilities literature and (b) the bottleneck of human subjects testing; and we suggest that recent methodological advances and discoveries could be harnessed to fundamentally rebalance our broad understanding of human talent toward a greater appreciation of specific abilities. We will base this argument on insights we have gained from researching face recognition ability. Work in our labs and others has recently established face recognition as an exceptionally specific ability (Wilmer et al., 2010, 2012; Wilhelm et al., 2010; Davis et al., 2011; Hildebrandt et al., 2011; Peterson and Miller, 2012; McGugin et al., 2012; Palermo et al., 2013).

To be clear, when we use the terms *specific*, *specific* *ability*, *specificity*, or *specifically* in this paper, we use them in their classic human variation sense to refer to performance that correlates little across individuals with general intelligence (Spearman, 1904; Carroll, 1993; Jensen, 1998). The term *specific* is frequently used differently in the experimental psychology and human neuroscience literatures. In these literatures, it refers neither to individual differences nor to general intelligence, but it is rather used as a shorthand for domain or process specificity (Gazzaniga, 2004). While studies of individual differences can and do effectively tackle questions of domain and process specificity (Wilmer, 2008), here we focus on the more basic question of whether an ability dissociates from (is specific relative to) general intelligence.

In the first section below, we briefly review the evidence that face recognition varies specifically across individuals. In the second section, we examine two illuminating false starts whereby well-resourced efforts to measure face recognition ability misinterpreted promising evidence for its specificity. These false starts demonstrate how easily a specific ability can be overlooked. In the third and final section, we identify three key factors that fueled the recent discovery that face recognition ability is specific and that, we believe, could likewise fuel the discovery of further specific abilities. These factors were: incorporation of priorities, discoveries, and techniques from experimental psychology and cognitive neuroscience; the development and validation of an excellent test; and a powerful internet-enabled Citizen Science approach to investigating human variation.

## Face recognition varies specifically across individuals

The core evidence that face recognition varies specifically across individuals comes from two complementary sources. The first source is face recognition’s dissociations from other, more general cognitive abilities; the second, equally-critical source is the robust associations observed among assessments that measure face recognition ability in very different ways.

Face recognition, as measured by the widely-used Cambridge Face Memory Test (CFMT; Duchaine and Nakayama, 2006), dissociates strongly from more general abilities. It dissociates almost completely from standardized IQ tests. To date, its mean reported correlation with such IQ tests, weighted by sample size and corrected for range restriction in the IQ tests, is 0.01 (Davis et al., 2011; Peterson and Miller, 2012; Palermo et al., 2013). Face recognition, as measured by the CFMT, also dissociates surprisingly strongly from other recognition abilities. It shares a mere 3% of its variation with the recognition of word pairs (*n* = 3003; 95% CIs 2–4%; Wilmer et al., 2010, 2012); and even *within* the realm of visual recognition, it shares only 7% of its variation with the recognition of abstract art images (*n* = 4475; 95% CIs 5–8%; Wilmer et al., 2010, 2012).

These pervasive dissociations from other abilities are not a result of poor measurement. Not only is the CFMT as reliable per unit time as the most widely-used IQ test (Wechsler, 2008; Wilmer et al., 2010), but it correlates well with tests that measure face identity processing in quite different ways. Two such tests are the Cambridge Face Perception Test (CFPT), which correlates 0.60 with the CFMT (*n* = 124; Bowles et al., 2009), and the Cambridge Famous Faces Memory Test (CFFMT), which correlates 0.52 with the CFMT (*n* = 1219; Wilmer et al., 2010, 2012).

The CFPT and CFFMT differ from the CFMT in multiple ways. The CFMT assesses one’s ability to memorize a set of previously unfamiliar faces and then, shortly thereafter, recognize them among distractors. The CFPT, in contrast, assesses one’s ability to *rank* several faces by the similarity of their identity to a *simultaneously-viewed* “exemplar” face. The CFFMT, in further contrast, assesses one’s ability to *attach names or other identifying information to* celebrity faces *learned haphazardly over one’s lifetime*.

The CFMT, CFPT, and CFFMT thus differ starkly in both the task being performed (from visual matching in the CFPT to recognition in the CFMT to recall in the CFFMT) and the duration over which faces must be remembered (from milliseconds in the CFPT to minutes in the CFMT to years or decades in the CFFMT), making their robust intercorrelations a powerful demonstration of valid measurement.

Finally, and perhaps most impressive of all, the CFMT correlates 0.37 with a person’s self-rating with the single statement “I can recognize famous celebrities in photos or on TV” (*n* = 190; 95% CI 0.24–0.49; Wilmer et al., 2010). This is substantially larger than the average 0.15 correlation found between objective and self-report measures of memory abilities in a major meta-analysis of 24,897 individuals tested across 169 studies (Beaudoin and Desrichard, 2011).

Associations like these between CFMT and CFPT, CFFMT, and self-reported recognition ability critically distinguish specificity from invalid measurement. As we will see below, such associations, as a counterpoint to face recognition’s persistent dissociations, were the missing piece in prior face recognition ability research.

In addition to being specific, human variation in face recognition is highly heritable (Wilmer et al., 2010). This combination of specificity and heritability is rare (Wilmer et al., 2010). Indeed, so consistently has specificity traded off against heritability in past research that a recent behavioral genetic theory, the “generalist gene” theory, posited that most or all cognitive variation results from the same set of genes (Kovas and Plomin, 2006). A major exception to the generalist gene theory (Wilmer et al., 2010; Plomin et al., 2013), face recognition’s heritability demonstrates that different sets of genes contribute independently to human cognitive ability. Given the example of face recognition, it is worth considering not only how many other specific abilities exist, but also whether any of them are as strongly heritable as face recognition.

In sum, face recognition, at least when measured via the CFMT, is exceptionally specific. Moreover, it is rare among specific abilities for its high heritability. In the next section, by examining two earlier false starts in the valid measurement of face recognition ability, we illustrate barriers to the discovery of its specificity that could bear importantly on the search for further specific abilities.

## Two false starts in the measurement of face recognition ability

In this section, we will recount two major efforts to assess face recognition ability. These efforts, begun nearly 70 years apart, are among history’s most concerted efforts to measure any social ability (Kihlstrom and Cantor, 2000; Wilmer et al., 2012). In each case, initial promising evidence for face recognition’s specificity was misinterpreted as invalid measurement, and development of the test in question was abandoned. These missed opportunities to examine the specific ability of face recognition seem unlikely to us to be isolated examples. The lessons learned from these missed opportunities may therefore provide valuable information on where to search for additional specific abilities.

The first false start in the measurement of face recognition ability involved the George Washington Social Intelligence Test (GWSIT), developed in the late 1920s (Hunt, 1928). The GWSIT consisted of six subtests, two of which involved faces. A face recognition subtest assessed the ability to learn the names for a set of twelve novel target faces; then, presented with a larger group of faces, one was required to pick out the target faces and recall their names. The second subtest involving faces assessed the ability to label the mental states of faces based on their expression. The remaining four subtests verbally assessed other aspects of social knowledge and social judgment (Hunt, 1928).

The initial validation study for the GWSIT clearly showed that, though none of its subtests correlated particularly highly with each other (maximum *r* = 0.44), the face recognition subtest dissociated most strongly of all from the other subtests (mean *r* = 0.22; Hunt, 1928). On the basis of these dissociations, as well as a dissociation from a measure of general intelligence, Hunt (1928) presciently suggested that “the special ability of being able to recognize [faces] is relatively independent of pure ‘brains”’.

What happened next is telling. Surprisingly, at least in hindsight, the promising evidence that the GWSIT provided for face recognition’s specificity was not eagerly pursued. Quite to the contrary, the GWSIT was roundly criticized for failing to measure a unitary social ability. That is, it was criticized (a) because its subtests dissociated strongly from each other; and (b) because the small amount of overlap between its subtests was ultimately attributed to general intelligence (Thorndike, 1936; Thorndike and Stein, 1937). On this basis, the GWSIT rapidly fell out of favor as a research instrument (Kihlstrom and Cantor, 2000). Moreover, a mere two decades after it was introduced, the GWSIT was cited, in what would soon become the classic paper on test validity, as the classic example of an invalid test (Campbell and Fiske, 1959). In sum, far from inspiring further research, face recognition’s clear and persistent dissociations from other abilities were the core inspiration for the rejection of the GWSIT as a valid ability measure.

Lest one be tempted to write off the rejection of the GWSIT as an isolated historical event, let us move forward nearly 70 years to a second, remarkably similar story. This story involved the third edition of the highly influential Wechsler Memory Scale (WMS-III), introduced in 1997 (Wechsler, 1997). The WMS-III added, for the first time in the WMS’s history, a face recognition subtest. This subtest assessed the ability to memorize a set of faces and then classify a subsequent series of faces as “old” (seen before) vs. “new” (not seen before) (Wechsler, 1997). As with the GWSIT, the WMS-III’s face recognition subtest dissociated persistently from other measures. These other measures included the WMS’s own verbal and visual recognition subtests (Wechsler, 1997; Millis et al., 1999; Holdnack and Delis, 2004). Again, such dissociations were viewed as a liability rather than a virtue. The face recognition subtest was criticized for its dissociations (Millis et al., 1999; Holdnack and Delis, 2004), and it was dropped from the WMS-IV (Wechsler, 2009).

Seven decades apart, the story was the same. Face recognition’s dissociations fueled a presumption of invalid measurement and an abandonment of measures, with remarkably little work aimed at disentangling specificity from invalid measurement by examining correlations across diverse measures of face recognition ability. The persistence with which face recognition was overlooked in these cases illustrates a blind spot for specificity that we believe is broadly characteristic of traditional test development practices in the human ability literature.

## Face recognition as a model in the search for further specific abilities

We will now discuss three key factors that fueled the recent discovery that face recognition varies specifically across individuals, and that could plausibly fuel the discovery of further specifically varying abilities. These factors were: incorporation of priorities, discoveries, and techniques from experimental psychology and cognitive neuroscience; the development and validation of an excellent test; and a powerful internet-enabled Citizen Science approach to investigating human variation.

In contrast to the human ability literature’s capacity to overlook dissociations, the cognitive neuroscience and experimental psychology literatures have, throughout their history, actively sought out dissociations. A remarkable aspect of the WMS story is that its face recognition subtest was introduced the same year, 1997, as major reports of face-selective activation in the human fusiform face area (FFA; see also Sergent et al., 1992; Kanwisher et al., 1997; McCarthy et al., 1997). Simultaneously, the WMS’s dissociations inspired disappointment and rejection, while the FFA’s dissociations inspired excitement and follow-up work. Indeed, the FFA’s dissociations, along with other key neural and cognitive dissociations, have played a central role in solidifying the status of face processing as a major model system in studies of mind and brain. Such different reactions to evidence for dissociation are instructive when considering where to look for specific abilities. Perhaps equally valuable inspiration on where to look could be derived from the orphan tests of human abilities research (tests that were reliable yet abandoned due to their persistent dissociations) and the core dissociable model systems of cognitive neuroscience and experimental psychology.

As illustrative examples of ability domains that could plausibly contain additional specific abilities, consider social cognition, navigation, and dynamic visual perception. In the case of social cognition, the dissociations produced by the GWSIT raise the possibility that additional specific social abilities may exist (Hunt, 1928; Kihlstrom and Cantor, 2000; see also Mayer et al., 2008), and several aspects of social cognition, including theory of mind and joint attention, have been associated with distinct neural areas (Saxe, 2006). Navigation and dynamic visual perception, too, each involve well-defined neural areas (Epstein and Kanwisher, 1998; Newsome and Pare, 1988), and appear to dissociate from at least some general abilities (Hegarty et al., 2006; Wilmer and Nakayama, 2007). These are merely a few illustrative examples of the many domains in which orphan tests and/or functional or neural dissociations exist. We expect that there exist tens or hundreds of additional areas were such evidence is compelling enough to consider initiating a search for specific abilities.

The recent discovery of face recognition’s specificity owes much to the careful development of a single, high-quality test: the CFMT (Duchaine and Nakayama, 2006). Ironically, it was the cognitive neuroscience and experimental psychology literatures, not the human abilities literature, that inspired the development of the CFMT (Duchaine and Nakayama, 2006). The CFMT’s development drew primarily from three scientific areas. First, it drew from the stimulus-control techniques of visual psychophysics to produce well-controlled stimuli. Second, it drew from the dissociation-focused manipulations of cognitive neuroscience and experimental psychology to achieve an effective isolation of face processing mechanisms. Third, it drew from the practical test design methods of patient-based neuropsychological testing to minimize its demands on test-takers’ general cognitive resources, including their capacity to attend, interpret, and problem-solve (taxing such general resources likely increases a test’s reliance on general intellectual ability) (Duchaine and Nakayama, 2006).

The exceptional specificity of face recognition, as measured by the CFMT, is a case study in the value of incorporating the priorities, discoveries, and techniques of cognitive neuroscience and experimental psychology into efforts to measure human ability. Meaningful progress in the isolation of specific abilities, however, additionally requires a combination of rigorous psychometrics and access to the large, diverse samples of participants that enable iterative development, validation, and norming of high-quality tests.

Fortunately, we live at a time when the internet has opened up unprecedented opportunities for testing large samples. Resources such as Amazon Mechanical Turk^®^–an online clearing-house for small jobs where psychological research is increasingly conducted—enable the rapid recruitment and testing of large samples. Our own web-based work on face recognition and other abilities has been powered by our *Citizen Science* project TestMyBrain.org (Germine et al., 2012). As with other citizen science initiatives (Bonney et al., 2009), TestMyBrain.org seeks to actively collaborate with the general public to answer scientific questions. At TestMyBrain.org, we make high-quality tests freely available via the web, and participants complete these tests to learn about themselves. We then aggregate data across participants to further refine the tests we offer and to answer scientific questions. Due to high public interest in self-discovery, the ease of participation across demographic groups, and the near-zero incremental cost of recruiting and testing each additional participant, our studies of face recognition have been able to rapidly collect high-quality data from many thousands of individuals of varied age, sex, occupation, and socioeconomic status (Wilmer et al., 2010, 2012; Germine et al., 2011a,b, 2012). Citizen science projects like TestMyBrain.org, as well as other large-scale internet-based testing projects like Mechanical Turk, provide the necessary throughput to capture specific abilities and examine their importance in our lives.

## Conclusions

Here, we have examined face recognition as a model specific ability. First, we reviewed the recent work that documents face recognition’s specificity. Second, we recounted two major false starts in the measurement of face recognition ability. These false starts reveal a capacity for specific abilities not only to be missed, but indeed, to be actively avoided by major test development efforts. Third, we discussed three key factors that contributed to the discovery that face recognition ability is specific and that, we believe, could serve as a compass for the discovery of further specific abilities. These factors were: incorporation of priorities, discoveries, and techniques from experimental psychology and cognitive neuroscience; the development and validation of an excellent test; and a powerful internet-enabled Citizen Science approach to investigating human variation. We suggest that the time is right for a renewed effort to investigate specific abilities, and that this effort can be guided by the model example of face recognition ability. By revealing relatively independent dimensions of human ability, such work would enhance our capacity to understand the uniqueness of individual minds.

## Conflict of interest statement

The authors declare that the research was conducted in the absence of any commercial or financial relationships that could be construed as a potential conflict of interest.
